# Supplementary material for: Aerobic exercise up-regulates Klotho to improve renal fibrosis associated with aging and its mechanism
Source: PLoS One. 2024 Sep 26;19(9):e0311055. doi: 10.1371/journal.pone.0311055 (PMC11426507; doi:10.1371/journal.pone.0311055)
Supplement: S1 Raw image — (PDF) [file pone.0311055.s001.pdf]

WB 1:Aerobic exercise improved renal impairment and renal fibrosis in aged mice

Repeat1 （As presented in the study）

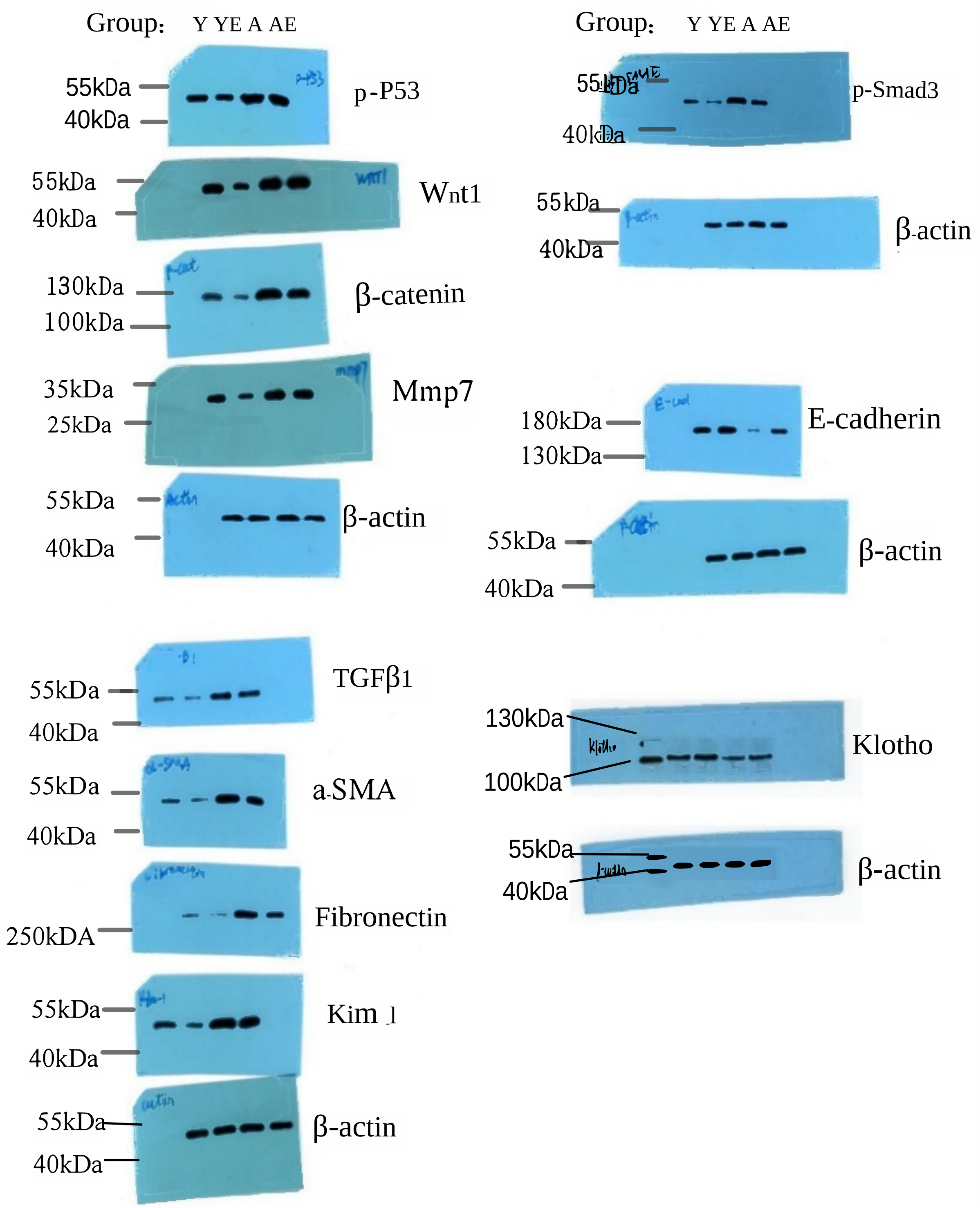

Repeat 2

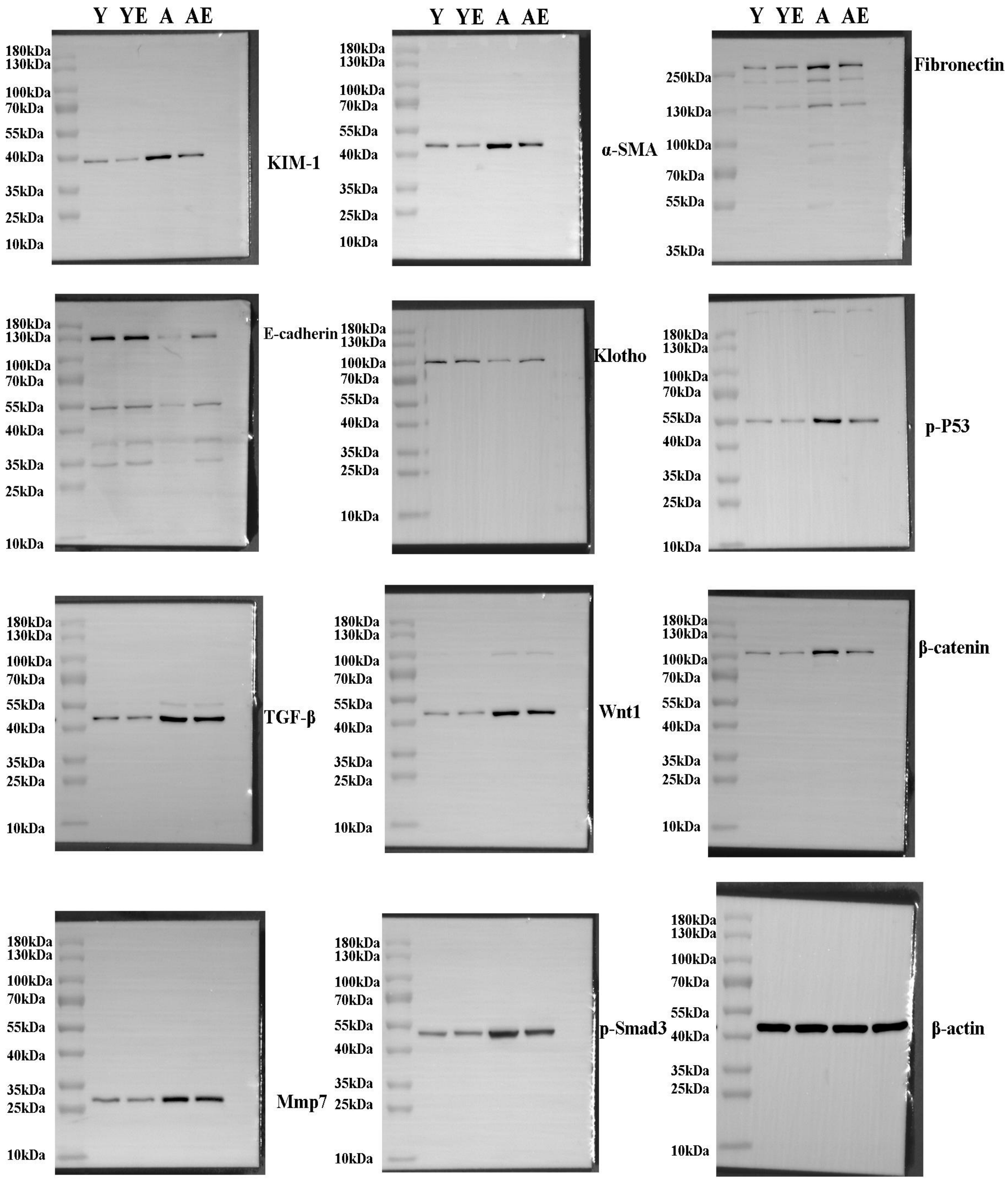

Y YE A AE
180kDa
130kDa
100kDa
70kDa
55kDa
40kDa
35kDa
25kDa
10kDa
KIM-1
Y YE A AE
180kDa
130kDa
100kDa
70kDa
55kDa
40kDa
35kDa
25kDa
10kDa
α-SMA
Y YE A AE
250kDa
130kDa
100kDa
70kDa
55kDa
35kDa
Fibronectin
180kDa
130kDa
100kDa
70kDa
55kDa
40kDa
35kDa
25kDa
10kDa
E-cadherin
180kDa
130kDa
100kDa
70kDa
55kDa
40kDa
35kDa
25kDa
10kDa
Klotho
180kDa
130kDa
100kDa
70kDa
55kDa
40kDa
35kDa
25kDa
10kDa
p-P53
180kDa
130kDa
100kDa
70kDa
55kDa
40kDa
35kDa
25kDa
10kDa
TGF-β
180kDa
130kDa
100kDa
70kDa
55kDa
40kDa
35kDa
25kDa
10kDa
Wnt1
180kDa
130kDa
100kDa
70kDa
55kDa
40kDa
35kDa
25kDa
10kDa
β-catenin
180kDa
130kDa
100kDa
70kDa
55kDa
40kDa
35kDa
25kDa
10kDa
Mmp7
180kDa
130kDa
100kDa
70kDa
55kDa
40kDa
35kDa
25kDa
10kDa
p-Smad3
180kDa
130kDa
100kDa
70kDa
55kDa
40kDa
35kDa
25kDa
10kDa
β-actin

Repeat 3

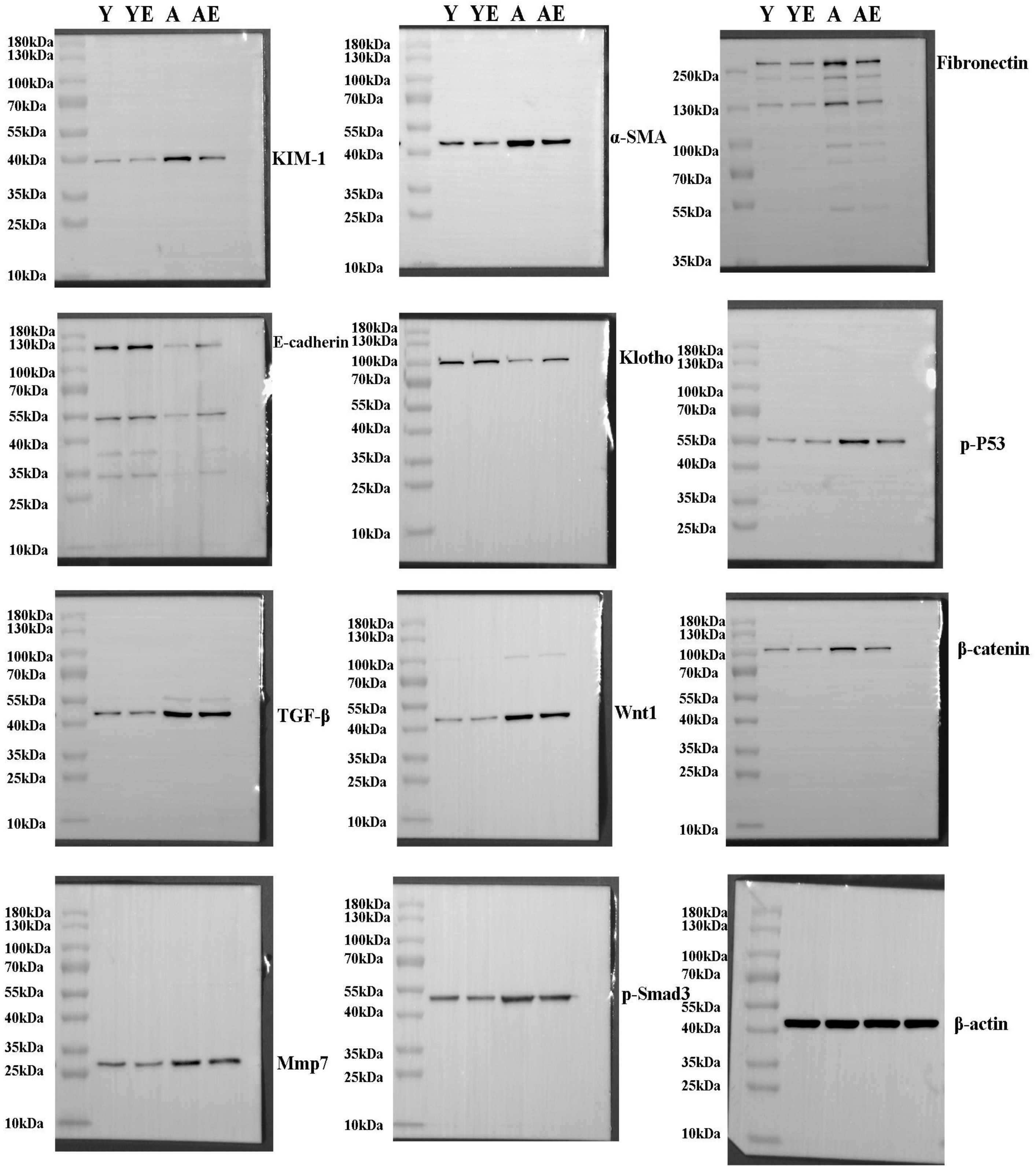

Y YE A AE
Y YE A AE
Y YE A AE
180kDa
130kDa
100kDa
70kDa
55kDa
40kDa
35kDa
25kDa
10kDa
KIM-1
α-SMA
Fibronectin
250kDa
E-cadherin
Klotho
p-P53
TGF-β
Wnt1
β-catenin
Mmp7
p-Smad3
β-actin

WB 2: Rescue effect of aerobic exercise in Klotho-inhibited mice

Repeat 1 （As presented in the study）

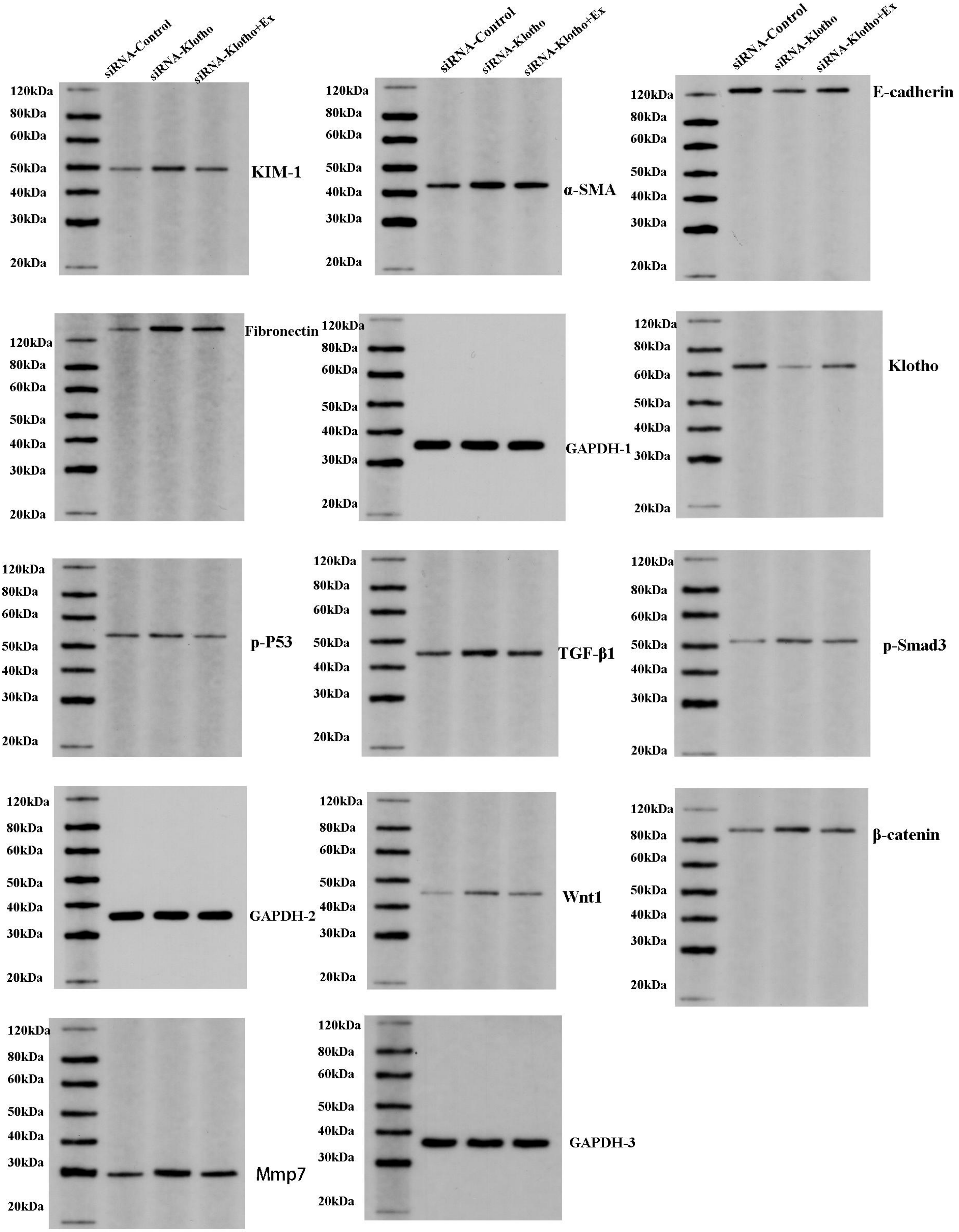

Repeat 2

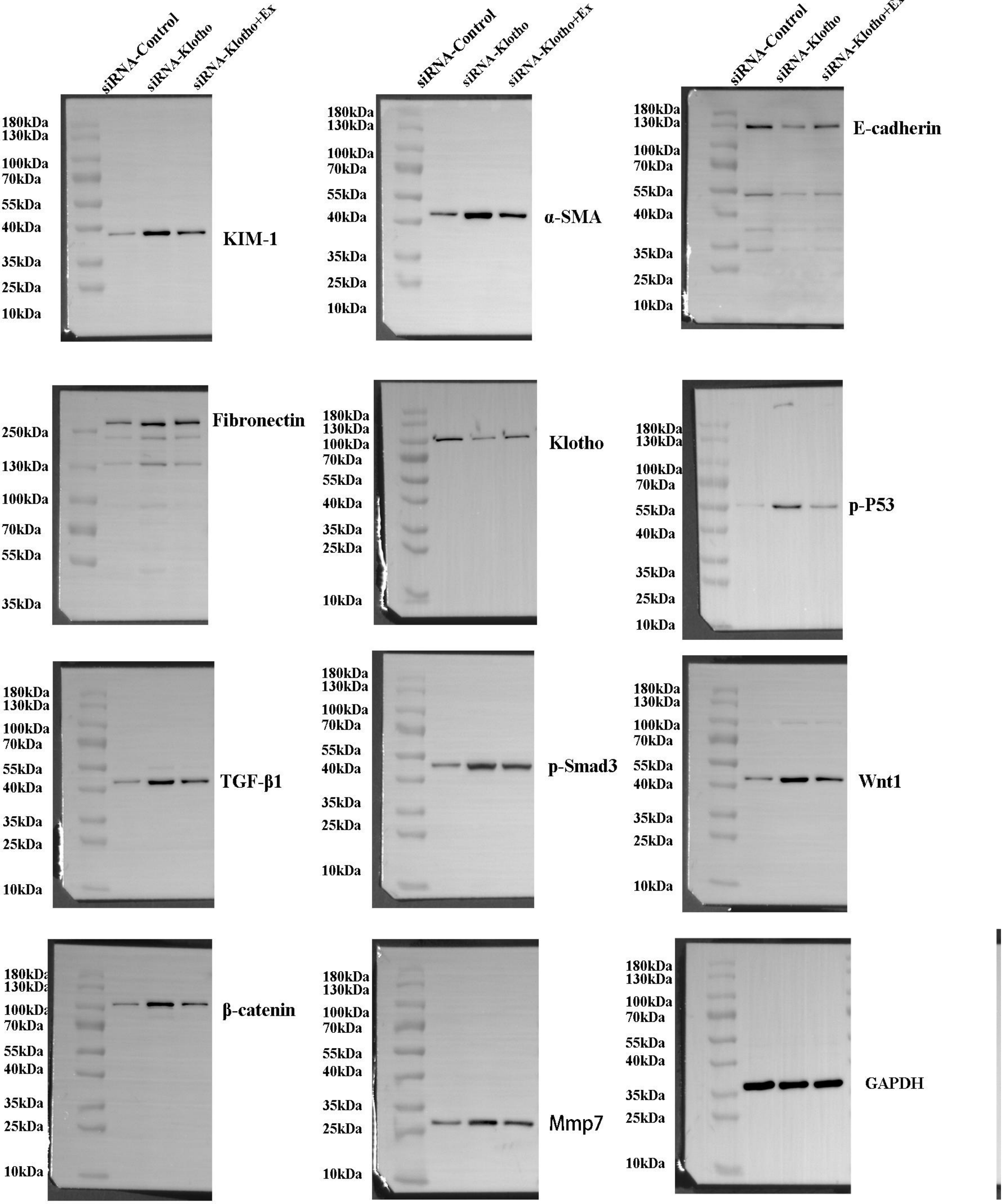
siRNA-Control
siRNA-Klotho
siRNA-Klotho+Ex
KIM-1
α-SMA
E-cadherin
Fibronectin
Klotho
p-P53
TGF-β1
p-Smad3
Wnt1
β-catenin
Mmp7
GAPDH
250kDa
180kDa
130kDa
100kDa
70kDa
55kDa
40kDa
35kDa
25kDa
10kDa

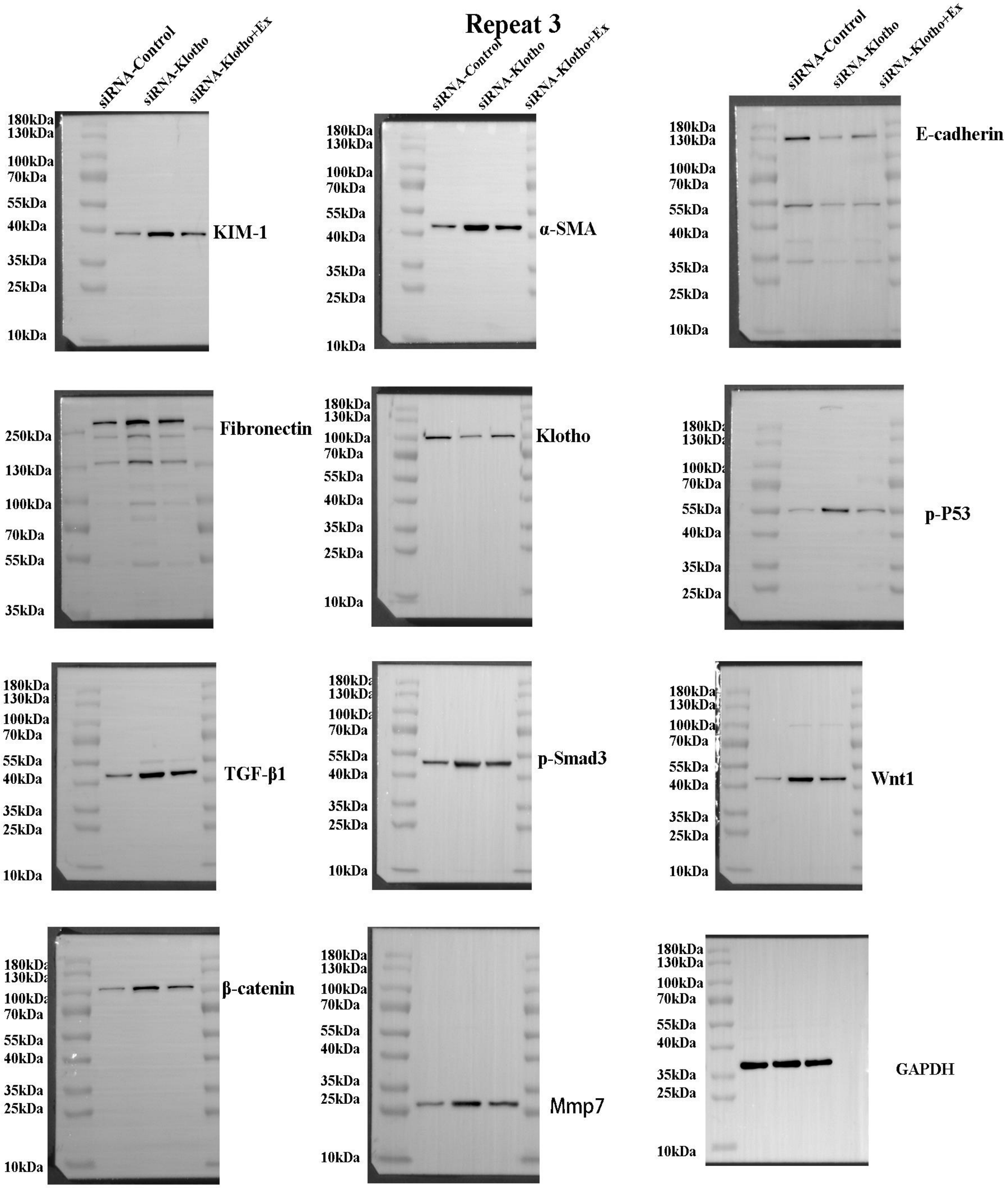
Repeat 3
siRNA-Control
siRNA-Klotho
siRNA-Klotho+Ex
KIM-1
α-SMA
E-cadherin
Fibronectin
Klotho
p-P53
TGF-β1
p-Smad3
Wnt1
β-catenin
Mmp7
GAPDH
250kDa
180kDa
130kDa
100kDa
70kDa
55kDa
40kDa
35kDa
25kDa
10kDa
