# Supplementary material for: Aerobic exercise up-regulates Klotho to improve renal fibrosis associated with aging and its mechanism
Source: PLoS One. 2024 Sep 26;19(9):e0311055. doi: 10.1371/journal.pone.0311055 (PMC11426507; doi:10.1371/journal.pone.0311055)
Supplement: S1 File — (DOCX) [file pone.0311055.s002.docx]

**Table 1. Training protocol**

| Week | Day | Velocity  (m·min-1) | Duration (min) | Slope (%) |
| --- | --- | --- | --- | --- |
| adaptive exercise | 1-2 | 8 | 10 | 0 |
|  | 3-4 | 11 | 20 | 3 |
|  | 5-6 | 14 | 30 | 6 |
|  | 7 | Rest | | |
| 1-12weeks | 1-3  5-6 | 8 | 5 | 6 |
|  |  | 10 | 2 | 6 |
|  |  | 12 | 2 | 6 |
|  |  | 14 | 30 | 6 |
|  |  | 5 | 2 | 6 |

**Table 1. Training protocol**

YE and AE groups were trained on an animal treadmill with accumulating time and intensity for six days to get accustomed to the exercise. Thereafter, mice were trained on a treadmill for 41 minutes at an incline of 6% , 5 days/week for 12 weeks.

**Table. 2 Primer sequences used in the study**

| Gene | Specie | Forward (F) Primer Sequence | Reverse (R) Primer Sequence |
| --- | --- | --- | --- |
| miR-34a | Mice | 5-TGCGCTGGCAGTGTCTTAGCTG-3 | |
| a-SMA | Mice | F: 5-CACCACTGAACCCTAA-3 | R: 5-GTCCAGCACAATACCA-3 |
| Kim-1 | Mice | F:5-AGTGGAGATTCCTGGATGGTTT-3 | R: 5-GTAGCTGTGGGCCTTGTAGTTG -3 |
| E-cadherin | Mice | F: 5-CAGGTCTCCTCATGGCTTTGC-3 | R:5-CTTCCGAAAAGAAGGCTGTCC-3 |
| Fibronectin | Mice | F:5-TGGTGTTTTCCACGGATGCT -3 | R:5- GTGTGCCCACTGCTGACTTA-3 |
| klotho | Mice | F:5-ACTACGTTCAAGTGGACACTACT-3 | R:5-GATGGCAGAGAAATCAACACAGT-3 |
| TGF-β1 | Mice | F: 5-CCGCAACAACGCCATCTAT-3 | R:5-GTCAGCAGCCGGTTACCAA-3 |
| Wnt 1 | Mice | F:5-GATTTTGGTCGCCTCTTT-3 | R:5-GGACATCCCGTGGCATTT-3 |
| β-catenin | Mice | F: 5-ATGGAGCCGGACAGAAAAGC-3 | R:5-CTTGCCACTCAGGGAAGGA-3 |
| Mmp7 | Mice | F:5-CTGGAAAACTCTCCCCCT-3 | R:5-TCCCCCAACTAACCCTCT-3 |
| GAPDH | Mice | F:5- TCAACGACCACTTTGTCAAGCTCA-3 | F:5- GCTGGTGGTCCAGGGGTCTTACT-3 |

**Table. 3 Serum levels of BUN and Cr**

**A.**

|  | **Y** | **YE** | **A** | **AE** |
| --- | --- | --- | --- | --- |
| **BUN（mmol/L）** | 10.68±1.6 | 8.672±1.505 | 14.2±0.61 | 11.89±0.34 |
| **Cr（mmol/L）** | 61.16±4.37 | 53.28±2.308 | 70.37±3.688 | 64.28±2.971 |

**B.**

|  | **siRNA-control** | **siRNA-Klotho** | **siRNA-Klotho+Ex** |
| --- | --- | --- | --- |
| **BUN（mmol/L）** | 10.07 ±0.9663 | 12.33±1.319 | 10.22±0.7211 |
| **Cr（mmol/L）** | 50.63±2.375 | 63.56±1.764 | 58.3±1.984 |
